# Bioactive Properties of Instant Chicory Melanoidins and Their Relevance as Health Promoting Food Ingredients

**DOI:** 10.3390/foods12010134

**Published:** 2022-12-27

**Authors:** Sílvia Petronilho, Joana Navega, Carla Pereira, Adelaide Almeida, João Siopa, Fernando M. Nunes, Manuel A. Coimbra, Cláudia P. Passos

**Affiliations:** 1 Laboratório Associado para a Química Verde, Department of Chemistry, University of Aveiro, Campus Universitário de Santiago, 3810-193 Aveiro, Portugal; 2 Chemistry Research Centre-Vila Real, Department of Chemistry, University of Trás-os-Montes and Alto Douro, Quinta de Prados, 5001-801 Vila Real, Portugal; 3 CESAM—Centre for Environmental and Marine Studies, Department of Biology, University of Aveiro, Campus Universitário de Santiago, 3810-193 Aveiro, Portugal

**Keywords:** chicory, coffee, high molecular weight, inulin, phenolic compounds, antioxidant, antimicrobial, antidiabetic

## Abstract

Instant chicory is a caffeine-free brew worldwide consumed as a coffee substitute. Like coffee grounds processing, chicory roots suffer a roasting process, which may lead to the formation of high-molecular weight nitrogen-brown compounds, the melanoidins. It is hypothesized that similarly to coffee, chicory melanoidins have health promoting potential. In this work, the chemical composition and biological activity of chicory high molecular weight material (HMWM) was evaluated. The chicory HMWM is composed by 28.9% (*w*/*w*) of carbohydrates, mainly fructose-rich polysaccharides (18.7% *w*/*w*) and 5.7% (*w*/*w*) of protein, distinct from coffee. The phenolic compounds constituent of the HMWM were mainly present in glycosidically linked and condensed structures (0.9 g/100 g and 5.8 g/100 g), showing in vitro ABTS^•+^ scavenging (IC_50_ = 0.28 mg/mL) and ferric ion reducing capacity (ca. 11 µg Fe^2+^ eq/mg). Chicory HMWM revealed to be effective against Gram-positive bacteria, mainly *Staphylococcus aureus* and *Bacillus cereus,* although not so efficient as coffee. It also showed potential to inhibit α-glucosidase activity (15% of inhibition), higher than coffee HMWM, approaching acarbose activity that is used in type 2 diabetes *mellitus* treatment. Thus, chicory melanoidins, when used as a food ingredient, may contribute to an antioxidant diet and to prevent diabetes, while increasing the protective effects against pathogenic bacteria.

## 1. Introduction

Chicory, belonging to the *Cichorium* genus and the Asteraceae family, is a worldwide cultivated perennial plant, being its leaves, flowers, and roots well recognized in different food applications, as in salads and infusions [1,2]. From these plant parts, roasted chicory roots are by far the most consumed ones as a coffee substitute [3], mainly due to the caffeine-free and pleasant taste of the resulting brew [4,5]. Chicory roots are a source of health promoting biomolecules, namely inulin, a non-reducing polysaccharide of fructose with (β2→1) glycosidic-linkages partly terminated by glucose monomers [1]. Inulin accounts for up to 45% of the total root constituents on a dry weight basis [6]. It has been related to a wide variety of health promoting properties, including prebiotic [7,8], bone mineralization [9], and anti-tumor [10], among other activities [11]. Chicory roots are also a source of phenolic compounds, such as caffeic, coumaric, and chlorogenic acids [6], whose content is commonly linked to the antioxidant [12,13] and antibacterial [14,15] potential of chicory. The antimicrobial activity of chicory roots can also derive from sesquiterpene lactones, like 8-deoxylactucin and 11β,13-dihydrolactucin [16].

To be consumed as a coffee substitute brew, the roots of chicory are normally submitted to drying, roasting (140–180 °C for 20–60 min), grinding, and spray-drying [5], which is quite similar to the coffee beans thermal process [17], although using lower temperatures and extended times. During this process, the chemical composition of the chicory roots is changed due to the degradation and/or transformation of some of the compounds identified in the raw material. Inulin and most of the amino acids present in chicory roots are largely degraded during the roasting process, whereas monosaccharides, such as glucose and fructose, decrease at the beginning and then increase until the dark roasting stage (173 °C, for 55 min) [18]. These changes are probably due to the occurrence of reactions between reducing sugars and free amino groups, leading to the formation of a variety of products [19]. Melanoidins are the final product of these Maillard reactions and are generically defined as high molecular weight nitrogenous brown colored compounds [20,21], composed by moieties rich in polysaccharides, proteins, and phenolic compounds, covalently linked to brown compounds [22]. Coffee [17,23] and barley [21,24] melanoidins are the most studied ones, and have been related to antioxidant, anti-inflammatory [17,25], dietary fiber effect and prebiotic capacity [21], among other activities [26,27]. Taking into consideration the relevance of instant chicory in the global market, this work aims to characterize the high molecular weight material (HMWM) of this coffee substitute and evaluate its biological effects using in vitro assays.

## 2. Materials and Methods

### 2.1. Samples and Chemicals

Instant soluble chicory (Biocop, 100% soluble chicory, Amcore Balance, Barcelona, Spain) was purchased in the market. For comparison, a commercial instant soluble coffee (Nescafé classic, 100% natural soluble coffee with a mixture of Arabica and Robusta varieties, Nestlé Portugal SA., Linda-a-Velha, Portugal) was also used.

For carbohydrate analysis, sulfuric acid (H_2_SO_4_, 98%, Biochem Chemopharma, Cosne-Cours-sur-Loire, France), sodium borohydride (NaBH_4_, >95%, Fischer Chemical^TM^, Waltham, Middlesex, MA, USA), acetic anhydride (C_4_H_6_O_3_, ≥99%, Carlo Erba Reagents, Cornaredo, MI, Italy), 1-methylimidazole (C_4_H_6_N_2_, ≥99%, Sigma-Aldrich, Madrid, Spain), hydrochloric acid (HCl, 37%, Sigma-Aldrich, Madrid, Spain), acetic acid glacial (C_2_H_4_O_2_, ≥99%, Carlo Erba Reagents, Cornaredo, MI, Italy), dichloromethane (CH_2_Cl_2_, 99.8%, Fischer Chemical^TM^, Waltham, Middlesex, MA, USA), and 2-deoxyglucose (C_6_H_12_O_5_, ≥99%, Sigma-Aldrich, Madrid, Spain) were used. For phenolic analysis, Folin–Ciocalteu reagent, sodium carbonate (Na_2_CO_3_, ≥99.5%), and gallic acid (≥99%, HPLC) were from Sigma-Aldrich, Madrid, Spain. Also, sodium hydroxide (NaOH) and ethylenediaminetetraacetic acid (EDTA) from Panreac, Barcelona, Spain, hydrochloric acid (HCl), methanol (≥99.9%, HPLC), ethanol (≥99.9%, HPLC), veratric acid ((CH_3_O)_2_C_6_H_3_CO_2_H, ≥99%), and diethyl ether ((CH_3_CH_2_)_2_O, ≥99%) from Sigma-Aldrich, Madrid, Spain, and zinc powder (Fisher Chemical^TM^, Waltham, Middlesex, MA, USA) were used. Caffeic acid, ferulic acid, *p*-coumaric acid, catechol, salicylic acid, 2,3-dihydroxybenzoic acid, resorcylic acid, benzoic acid, 3,4-dihydroxybenzoic acid, 4-hydroxybenzoic acid, and gentisic acid were from Fisher Chemical^TM^, Waltham, Middlesex, MA, USA. For antioxidant activity analysis, [2,2′-azino-bis(3-ethylbenzothiazoline-6-sulphonic acid)] (ABTS, >98%), ascorbic acid (>98%), 2,4,6-tripyridyl-s-triazine (TPTZ, ≥98%), ferric chloride hexahydrate (FeCl_3_·6H_2_O, ≥97%), and iron sulfate (FeSO_4_, ≥99%) were from Sigma-Aldrich, Madrid, Spain. For antibacterial analysis, tryptic soy agar (TSA, Liofilchem, Roseto degli Abruzzi, Italy) and phosphate buffered saline (PBS, Sigma-Aldrich, St. Louis, MO, USA) were used. For the antidiabetic analysis, α-glucosidase (from *Saccharomyces cerevisiae*, 26% of protein, 179 U/mg of protein), *p*-nitrophenyl glucopyranoside (*p*NPG, ≥99%), sodium carbonate (Na_2_CO_3_, ≥99.5%), and acarbose (C_25_H_43_NO_18_, ≥95%) were from Sigma-Aldrich, Madrid, Spain.

### 2.2. Isolation of HMWM from Instant Chicory

Chicory HMWM was isolated after dissolving 20 g of chicory powder in 300 mL of water at 80 °C, for 20 min. After cooling at 4 °C and decanted, the infusion was dialyzed (4 °C, 12 kDa cut-off membrane (Medicell Membranes Ltd.; London, UK), 10 water renewals). Then, the retentate was freeze-dried to obtain the brown color (melanoidins) of high molecular weight material (HMWM). The same procedure was performed using instant coffee.

### 2.3. Characterization of HMWM of Instant Chicory

The isolated HMWM fraction from instant chicory was characterized in terms of its sugar content and composition, protein content, and total phenolic content and profile. The melanoidins content was estimated based on the brown color measurement (K_mix,405 nm_) and Melanoidin Browning Index (MBI). The same procedures were used for instant coffee.

#### 2.3.1. Carbohydrate Analysis

The neutral sugar content and composition were determined after acid hydrolysis, sugar residues derivatization to alditol acetates, and analysis by gas chromatography-flame ionization detection (GC-FID). For the acid hydrolysis, it was used 1 M H_2_SO_4_, at 100 °C, during 2.5 h [20]. After hydrolysis, it was performed a reduction with NaBH_4_ (15% *w/v* in 3 M NH_3_, 1 h, 30 °C) and acetylation with acetic anhydride in the presence of 1-methylimidazole (30 min, 30 °C). The 2-deoxyglucose was used as the internal standard [28,29]. The neutral sugar analysis was performed using two independent sample aliquots. In chicory sample, fructose was quantified as the sum of mannitol and glucitol using the ratio of the fructose epimerization during the reduction step [30]. Uronic acids were quantified, in duplicate, by the 3-phenylphenol colorimetric method after acid hydrolysis (1 M H_2_SO_4_, 100 °C, 1 h) of the sample and using galacturonic acid (GalA) as standard [31].

#### 2.3.2. Protein Analysis

The protein content was estimated through the determination of total nitrogen by elemental analysis in a Truspec 630–200-200 elemental analyzer (St. Joseph, Berrien, MI, USA) with a TDC detector (two independent aliquots per sample). The nitrogen content was converted to the estimation of protein (%, *w*/*w* dry sample) employing the 6.25 conversion factor [32]. For coffee HMWM the conversion factor was 5.5 [33].

#### 2.3.3. Phenolic Analysis

The total phenolic content was determined by Folin–Ciocalteu method [34]. Briefly, each sample (15 μL) was mixed with 15 μL of Folin reagent, 60 μL of distilled water, and 150 μL of sodium carbonate (7%). After incubation (60 °C, 30 min), the absorbance was measured at 725 nm. A calibration curve for gallic acid was build (5 to 250 μg/mL). Total phenolic content was expressed as μg gallic acid equivalents (GAE)/mg of sample (dry weight) using three independent aliquots.

The phenolic profile of the HMWM, namely adsorbed, esterified, glycosidically linked, and condensed one, was determined in triplicate. Adsorbed compounds [35]: a solution of HMWM sample (5 mg/mL in 1 M NaCl) was analyzed by direct injection by reversed-phase High Performance Liquid Chromatography with Photodiode Array Detector (RP-HPLC-DAD), using a ThermoFisher Scientific Vanquish Core HPLC system (Waltham, Middlesex, MA, USA) and a C_18_ column (Purospher^®^ STAR RP-18 endcapped LiChroCART^®^, 250 mm × 4.6 mm, 5 µm particle size, Merck, Darmstadt, Germany). Esterified compounds: the HMWM sample was subjected to alkaline saponification [35]. Briefly, a NaOH solution (2 M), containing ascorbic acid (2% *w*/*w*) and ethylenediaminetetraacetic acid (20 mM), was added to the HMWM aqueous solution (12 mg/mL). After incubation (1 h, 30 °C), the mixture was acidified with HCl (5 M) and stored (2h, 4 °C). After centrifugation, the supernatant was analyzed by RP-HPLC. Glycosidically linked compounds: the HMWM sample was subjected to methanolysis [36]. Briefly, a methanolic HCl solution (0.5 M) was added to the HMWM sample (5 mg) and heated (90 °C, 24 h). Then, the sample was evaporated (40 °C) and the internal standard (0.1 mg/mL veratric acid in methanol) was added. The sample was resuspended in a methanol solution and analyzed by RP-HPLC-DAD. Condensed compounds: the HMWM sample was subjected to alkaline fusion as described by Coelho et al. [35], with minor modifications. Solid NaOH (1 g) and zinc dust (100 mg) were melted (350 °C) and the HMWM sample (5 mg) was added. Then, the mixture was cooled on ice, solubilized in HCl (6 M), and the internal standard (1 mg/mL of veratric acid in ethanol) was added. The sample was acidified with HCl (pH 1–2) and the mixture was extracted with diethyl ether. After solvent evaporation, the residue was analyzed by RP-HPLC-DAD, using an Ultimate 3000 HPLC (Dionex, Thermo, Waltham, Middlesex, MA, USA) and a PRP-1 column (150 mm × 4.1 mm, 3 µm particle size, Hamilton Company, Reno, NV HQ, USA). Analytical grade standards and the internal standard method was used for quantification.

#### 2.3.4. Brown Color Measurement and Melanoidin Browning Index (MBI) 

The brown color of the HMWM sample was spectrophotometrically evaluated through the determination of the specific extinction coefficient at 405 nm (K_mix,405 nm_), using different sample dilutions (0–1 mg/mL) [28,33]. The curve contained at least 7 measurements and three independent replicates per sample concentration was performed. The MBI was estimated by the ratio of the brown color measurement (K_mix,405 nm_) and the unknown material which was estimated by the difference between the identified polymeric material (sugars and protein content) and the unknown one [33,37], as represented in Equation (1).

(1) $$\mathrm{MBI}=\frac{K_{\mathrm{mix},405\;\mathrm{nm}}}{\left(100\;-\;\mathrm{unknown}\;\mathrm{material}\right)}$$

### 2.4. In Vitro Biological Potential of Chicory HMWM 

The biological potential of the chicory HMWM was assessed using in vitro assays for the antioxidant, antibacterial, and antidiabetic activities determination. For comparison, coffee HMWM was also evaluated in terms of these in vitro properties.

#### 2.4.1. Antioxidant Activity

ABTS radical cation scavenging method [38]: Briefly, ABTS [2,2′-azino-bis(3-ethylbenzothiazoline-6-sulphonic acid)] cation radicals (ABTS^•+^) solution (250 μL) was added to 50 μL of sample with concentrations ranging from 0.04 mg/mL to 1.1 mg/mL. The absorbances were read after 20 min, in the dark, at 734 nm, in triplicate. A standard curve was recorded using ascorbic acid (0–20 μg/mL), a natural antioxidant of chicory roots. The IC_50_ (sample concentration that led to 50% of inhibition) values were calculated by plotting the percentage of scavenged ABTS^•+^ as a function of sample concentration. The kinetic behavior of the samples was assessed though the determination of the time needed to reach the steady state at IC_50_ concentration (T_IC50_) calculated from the kinetic curve (three independent replicates). Besides, the antiradical efficiency (AE), to express the antioxidant capacity of the samples, was also achieved (Equation (2)) [39].

(2) $$\mathrm{AE}=\frac{1}{{\mathrm{IC}}_{50}}{\;\times \;T}_{\mathrm{IC}50}$$

Ferric ion reducing antioxidant power (FRAP) assay [40]: FRAP is a method that has been applied to evaluate the Fe^2+^ chelating ability of dietary melanoidins [41]. A FRAP solution was prepared by mixing 25 mL of acetate buffer (0.3 M, pH 3.6) with 2.5 mL of 2,4,6-tripyridyl-*s*-triazine (TPTZ, 10 mM in 40 mM HCl) and 2.5 mL of ferric chloride solution (20 mM) and 3 mL of distilled water. The sample (20 μL), in a concentration range of 1.25 mg/mL to 25 mg/mL, was added to 289 μL of FRAP solution and maintained at 37 °C, for 30 min. The absorbance was measured, at 595 nm, in triplicate. Based on a FeSO_4_ standard curve (0.1–1 mM), results were expressed as µg Fe^2+^ equivalent/mg sample (dry weight).

#### 2.4.2. Antibacterial Activity

The antibacterial effect of the HMWM was tested against the growth of *Staphylococcus aureus* (ATCC^®^ 6538), *Listeria monocytogenes* (NCTC^®^ 1194), and *Bacillus cereus* (ATCC^®^ 11768). Fresh bacterial cultures were inoculated in 30 mL of tryptic soy agar (TSA) at 37 °C, 170 rpm, for 18 h. Then, the bacterial cultures were diluted in phosphate-buffered saline (PBS) solution (pH 7.4), adjusted to 0.5 McFarland standard, which corresponds to 10^8^ colony forming units (CFUs) per mL. The bacterial inoculum (final concentration of 10^5^ CFU/mL) was added to both samples, obtaining the final concentrations of 0.39, 0.78, 1.56, 3.13, 6.25, 12.50, 25.00, and 50.00 mg/mL. A bacterial growth control with only bacterial inoculum was included. The bacterial suspensions were incubated at 37 °C, for 24 h. Then, the aliquots of bacterial suspensions were sampled, serially diluted in PBS, and plated, in triplicate, in TSA. After incubation (37 °C, 18 h), the CFUs were calculated, and the viable bacterial density was determined as log CFU/mL. The Minimum Inhibitory Concentration (MIC) values were taken as the lowest concentration of the samples showing no growth in the plates [42]. Three independent experiments per each sample type and concentration were done.

#### 2.4.3. Antidiabetic Activity

The antidiabetic activity was assessed, in triplicate, through the α-glucosidase inhibition activity assay [43]. Briefly, a solution of α-glucosidase (0.075 unit) was mixed with the sample (4 mg/mL). Then, 3 mM *p*-nitrophenyl glucopyranoside (*p*NPG), in phosphate buffer (67 mM, pH 6.8), was added and incubated (37 °C, 30 min). The reaction was stopped by adding Na_2_CO_3_ (0.1 M). The α-glucosidase activity was determined by measuring the *p*-nitrophenol (*p*NP) released from *p*NPG at 400nm. The results were expressed as percentage of α-glucosidase activity inhibition. For comparison, the same process was performed using acarbose, a synthetic oligosaccharide used as α-glucosidase inhibitor in the treatment of type 2 diabetes *mellitus*.

### 2.5. Statistical Analysis

The chemical characterization data was statistically evaluated by applying the student’s *t*-test with a level of significant difference of 95% and *p* < 0.05, using the “test t” tool of Excel 2016. Additionally, for multiple comparison analysis (antibacterial and antidiabetic activity), one-way ANOVA with 95% probability level was used using GraphPad Prism version 8 for Window (trial version GraphPad software, San Diego, CA, USA). 

## 3. Results and Discussion

The yield of chicory high molecular weight material (HMWM), obtained after dialysis and subsequent freeze-drying, was 14.6% (dry weight). For instant coffee the yield of HMWM was 13.7%, in line with literature for the HMWM of coffee infusions (12.3%) [25]. Both HMWM samples were then characterized in terms of their chemical composition, as well as bioactive potential using in vitro assays.

### 3.1. Characterization of HMWM of Instant Chicory

**Figure 1:**
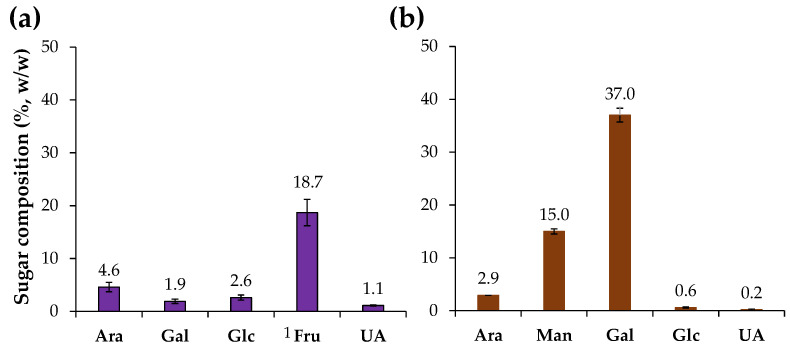
Sugar composition (%, *w*/*w*) of chicory (**a**) and coffee (**b**) high molecular weight material. ^1^ Fru was estimated as the sum of mannitol and glucitol using its epimerization ratio during the reduction step [30]. Sugar residues: Ara—arabinose, Man—mannose, Gal—galactose, Glc—glucose, Fru—fructose, and UA—uronic acids.

Chicory HMWM was analyzed for their neutral sugar and uronic acids content (Figure 1). The chicory HMWM was composed by 28.9% (*w*/*w*) of sugars (Table 1), mainly fructose (18.7%) derived from the inulin-rich composition of chicory roots [6], and small amounts of arabinose, glucose, galactose, and uronic acids (Figure 1), that can be related to the presence of pectin and other soluble polysaccharides [2]. A distinct sugar composition was determined for the coffee HMWM which was composed by 55.7% (*w*/*w*) of sugars (Table 1), mainly galactose (37%) and mannose (15%) and lower amounts of arabinose (Figure 1), being related to galactomannans and arabinogalactans present in coffee beans [44]. The sugar composition and content of the coffee sample was in line with literature (ca. 52% [25] to ca. 59% [21] *w*/*w* of sugars in instant coffee HMWM). Moreover, the chicory HMWM revealed to contain 5.7% of protein, while coffee HMWM had 12.2% of protein (Table 1). The protein content of coffee HMWM was in line with literature [25].

**Figure 2:**
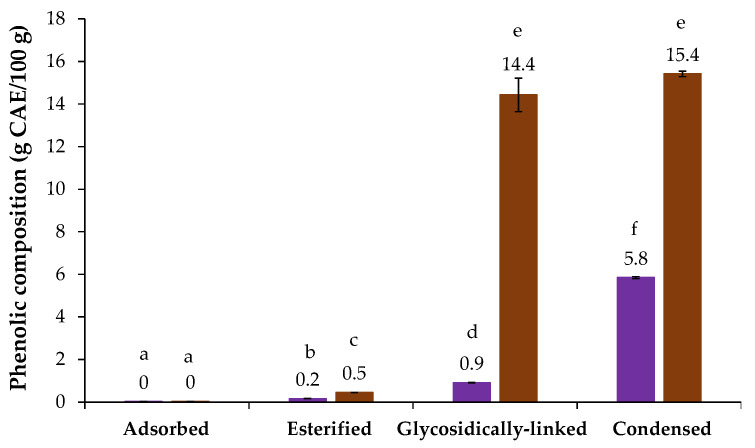
Phenolic composition (g caffeic acid equivalents (CAE)/100 g of sample) of chicory (purple bars) and coffee (brown bars) high molecular weight material. Lowercase letters refer to significantly different values (*p* < 0.05) among the samples.

The chicory HMWM presented 60.0 µg GAE/mg sample of phenolic compounds, as determined by the Folin–Ciocalteu assay (Table 1). The phenolic profile of chicory HMWM was present in Figure 2, where it can be observed that no adsorbed phenolic compounds were detected by direct RP-HPLC analysis of chicory, following the trend observed for coffee and barley melanoidins [21]. However, the alkaline saponification process allowed to determine 0.2 g caffeic acid equivalents (CAE)/100 g of esterified phenolic compounds in chicory HMWM, being caffeic acid (0.22 mg/g) the major esterified one (Table 2). On the other hand, the methanolysis and alkaline fusion approaches, allowed to release high amounts of potentially glycosidically linked and condensed phenolic compounds in chicory HMWM (0.9 and 5.8 g CAE/100 g, respectively, Figure 2). The condensed phenolic compounds value is near to the one determined for the HMWM of barley, another well-known coffee brew substitute [21]. The glycosidically linked phenolics released from chicory HMWM were mainly caffeic and ferulic acids (8.69 mg/g and 0.45 mg/g, respectively), while during the alkaline fusion, a total of 9 condensed phenolic compounds were released from chicory HMWM, especially 3,4-dihydroxybenzoic acid, catechol, and 4-hydroxybenzoic acid that were the ones determined in higher amounts (19.02 mg/g, 7.12 mg/g and 4.97 mg/g, respectively, Table 2). Besides the antioxidant properties linked to chicory phenolics roots [12,13], the 3,4-dihydroxybenzoic acid, the major condensed phenolic acid herein determined, has been shown potential to prevent gastric cancer [45]. 

The coffee HMWM presented 133.9 µg GAE/mg sample of total phenolic compounds, which was higher than chicory HMWM one (60.0 µg GAE/mg sample, Table 1). As can be observed in Figure 2, when compared to chicory sample, significantly higher amounts of esterified (0.5 g CAE/100 g), potentially glycosidically linked (14.4 g CAE/100 g) and condensed phenolic compounds (15.4 g CAE/100 g) were obtained for coffee HMWM, which was consistent with the results from the Folin–Ciocalteu method (Table 1). Moreover, a high amount of potentially glycosidically linked phenolic compounds from coffee HMWM, ca. 2 times higher when compared to literature [36], was observed. This was mainly due to the presence of aglycones of caffeic and ferulic acids (117.75 mg/g and 27.22 mg/g, respectively, Table 2). Different sources of coffee, proportion of Arabica and Robusta in the samples, and/or differences in roasting conditions could explain these differences [21,36].

Regarding the proportion of brown color compounds, the K_mix,405 nm_ value for the HMWM of instant chicory was 1.3 L/g/cm, while 1.1 L/g/cm was the value determined for coffee HMWM (Table 1). A similar K_mix,405 nm_ value was obtained for the HMWM of coffee brews [33]. This indicates that chicory had a high proportion of brown compounds, even higher than coffee HMWM. However, the contribution of the unknown material to the brown color of the sample, provided by the MBI value [33,37] (Table 1), showed that the HMWM of chicory (1.9) was 2 times lower than the MBI determined for coffee HMWM (3.8). The MBI value determined for the HMWM of coffee is in agreement with the one found in literature [25]. These results showed that the unknown material (melanoidins) present in the HMWM of chicory, accounting for 65% *w*/*w*, contributed to ca. 2 times less to the brown color of the sample than the one present in coffee HMWM, which only corresponded to 32% *w*/*w* of the sample. 

### 3.2. In Vitro Biological Activities of Chicory HMWM

#### 3.2.1. Antioxidant Activity

The antioxidant activity was evaluated by determining the chicory HMWM ability to scavenge ABTS^•+^ radical and to reduce the Fe^3+^ to Fe^2+^ (FRAP method). Then, the results were compared to coffee HMWM (Table 3). 

Regarding the antiradical activity, the concentration of chicory HMWM to inhibit 50% of ABTS (IC_50_) was ca. 0.3 mg/mL with a T_IC50_ of 47.05 min (Figure S1), thus exhibiting a slow kinetic behavior (>30 min) [39], and an antiradical efficiency (AE) of 5.95 × 10^−4^ mg/mL/min (Table 3). A higher antiradical efficiency (3.05 × 10^9^ mg/mL/min) was obtained for coffee HMWM with an IC_50_ value ca. 3 times lower (0.08 mg/mL) than chicory HMWM and a rapid kinetic behavior (<5 min, Figure S1) [39]. This suggests that coffee HMWM was more effective in reactions involving free radicals than chicory, which can be related to its higher phenolic content (Figure 2, Table 1 and Table 2). A similar trend was observed when considering coffee and barley melanoidins, where a ca. 3 times higher ABTS^•+^ scavenging activity was observed in melanoidin population from instant coffee [21]. Moreover, the distinct kinetic behavior of both samples should be considered when thinking in antioxidant ingredients, although the slower ones as of chicory HMWM (Table 3), can be useful to extend their antioxidant capacity during time. 

The ferric ion reducing capacity of chicory HMWM was ca. 11 µg Fe^2+^ eq/mg of sample, while the one of coffee HMWM was ca. 2 times higher (Table 3), explained by the higher phenolic compounds present in coffee HMWM (Figure 2, Table 1 and Table 2). Coffee melanoidins have already shown greater affinity toward Fe^2+^ ions when compared to barley and dark beer melanoidins due to its higher phenolic compounds amount [41].

#### 3.2.2. Antibacterial Activity

**Figure 3:**
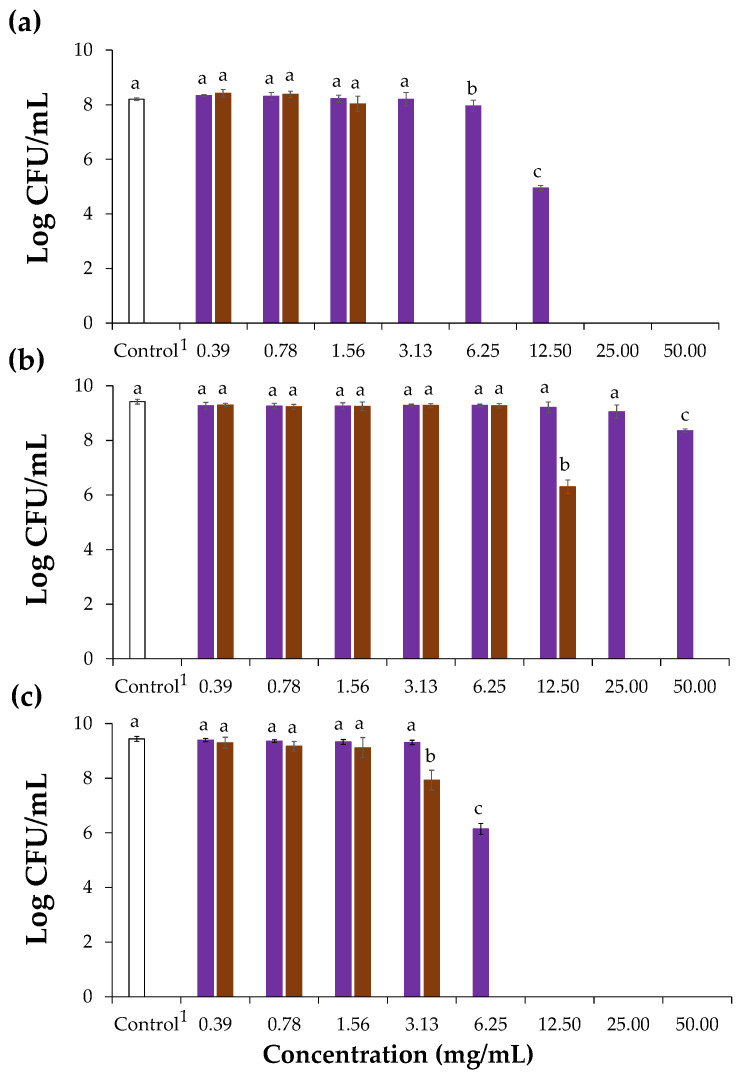
Effect of chicory (purple bars) and coffee (brown bars) high molecular weight material on the growth of *S. aureus* (**a**), *L. monocytogenes* (**b**), and *B. cereus* (**c**) represented as Log CFU/mL (CFU, colony forming units). ^1^ Control refers to assays with only the bacterial inoculum. Lowercase letters refer to significantly different values (*p* < 0.05) in relation to each corresponding control.

The antibacterial potential of chicory HMWM against Gram-positive and Gram-negative bacteria was firstly evaluated using *S. aureus* and *Escherichia coli*, respectively, using the inhibition zone method. Since the Gram-positive bacterium was found to be more sensitive than the Gram-negative one, where no diameter zone of inhibition was observed (Table S1), the antibacterial activity of chicory HMWM was only determined against three well known Gram-positive food contaminants, namely *S. aureus* (ATCC^®^ 6538), *L. monocytogenes* (NCTC^®^ 1194), and *B. cereus* (ATCC^®^ 11768) (Figure 3). The lower sensitivity of Gram-negative bacteria to different chicory plant-derived extracts was in line with literature [14]. It has been related to the extra hydrophilic outer membrane present in Gram-negative bacteria, consisting mainly of lipopolysaccharides that avoid the accumulation of phenolic compounds in the target cell membrane, the main responsible for antibacterial activity [46]. 

When compared to the control, chicory HMWM exhibited bacterial action at a range of 6.25 to 12.50 mg/mL, causing statistically significant decrease to 7.96 and 4.95 log CFU/mL, respectively against the bacterium *S. aureus*. Besides, at 6.25 mg/mL, chicory HMWM showed significant bactericidal effect against *B. cereus* (reduction to 6.14 log CFU/mL). However, for the bacterium *L. monocytogenes*, chicory HMWM proved to be ineffective even at a concentration of 50 mg/mL, although a significant reduction of 8.36 log CFU/mL was observed when compared to the control. This suggested that to suppress the growth of this bacterium, concentrations higher than 50 mg/mL are required (Figure 3). The inhibitory effect of chicory roots methanol extracts against Gram-positive bacteria was already reported, including against *S. aureus* and *B. cereus* [14], as well as of chicory roots ethanol-extracts against *S. aureus* and other *Bacillus* strains *(Bacillus subtilis* and *Bacillus thuringiensis*) [15]. The antibacterial activity herein determined for chicory HMWM can be ascribed to its phenolic compounds (Table 2). In fact, phenolics are able to interfere with Gram-positive bacteria cell membranes, affecting their permeability, releasing the intracellular constituents, and modifying the membranes’ functionality [47]. When compared to chicory HMWM, coffee totally suppressed *S. aureus*, *B. cereus*, and *L. monocytogenes* growth at 3.13 mg/mL, 6.25 mg/mL, and 25 mg/mL, respectively revealing that coffee HMWM had a greater capacity to inactivate the growth of Gran-positive bacteria (Figure 3). This higher antibacterial potential can be explained by the higher content on phenolic compounds determined in the HMWM of coffee when compared to the one from chicory (Figure 2, Table 1 and Table 2).

#### 3.2.3. Antidiabetic Activity

**Figure 4:**
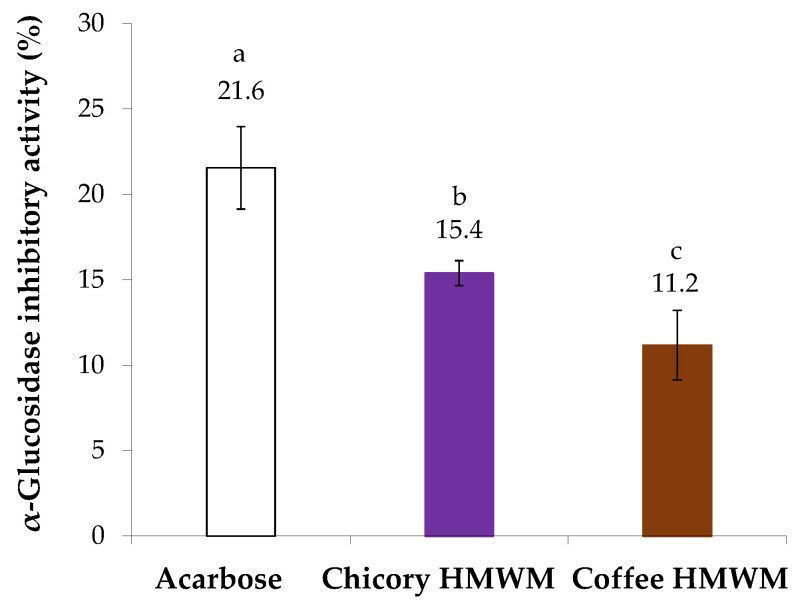
Inhibitory activity (%) against α-glucosidase of acarbose and chicory and coffee high molecular weight material (HMWM), at 4 mg/mL. Lowercase letters refer to significantly different values (*p* < 0.05) among samples.

The antidiabetic potential of chicory HMWM was determined considering its inhibitory ability against α-glucosidase, a digestive enzyme. For comparison purposes, acarbose, a synthetic oligosaccharide applied as an inhibitor of α-glucosidase in the treatment of type 2 diabetes *mellitus* was used (Figure 4). The inhibitory capacity against α-glucosidase of all samples under study was evaluated at the same concentration (4 mg/mL). Chicory HMWM exhibited 15.4% of inhibition of α-glucosidase activity, a value that, although lower, approached the value of acarbose standard (21.6% of inhibition). The antidiabetic potential of chicory HMWM can be related to its high fructose content derived from inulin (Figure 1). In fact, it was found that inulin derived from chicory water-soluble extracts (prepared at 70 °C, 50 min., constant stirring) caused a decrease in intestinal absorption of glucose, suggesting that products made of chicory (such as infusions) would be beneficial to healthy people, as well as to those with diabetes [48]. Thus, roasting conditions able to preserve the fructose-based compounds should be beneficial to potentiate the antidiabetic effect. When compared to chicory HMWM or even to acarbose, coffee HMWM exhibited a significantly lower inhibition of α-glucosidase activity (11% of inhibition). In line with the results herein obtained for coffee HMWM, it was found that, at 1.0 mg/mL, coffee melanoidins poorly inhibited the α-glucosidase activity [21].

## 4. Conclusions

Instant chicory accounted for 14.6% of brown colored HMWM, constituted by 28.9% of carbohydrates, mainly fructose from inulin-roots, and 5.7% of protein. After alkaline fusion, a high release of condensed phenolics was achieved (ca. 5.8 g/100g). When compared to coffee, a high proportion of brown compounds (K_mix,405 nm_ = 1.3 L/g/cm) was observed in chicory HMWM due to the presence of high amount of thermolabile carbohydrates. Nevertheless, chicory HMWM presented a relatively low MBI, that is, less brown melanoidins, possibly consequence of a mild roasting process when compared to coffee HMWM. The content in phenolic compounds may explain the chicory HMWM in vitro scavenging activity against ABTS^•+^ and ferric ion reducing antioxidant capacity, although lower than coffee HMWM. Moreover, chicory HMWM, by inhibiting the growth of Gram-positive bacteria, mainly *S. aureus* and *B. cereus*, have demonstrated antibacterial capacity. However, contrarily to coffee HMWM, chicory did not seem to have effect against *L. monocytogenes*. Besides, chicory HMWM showed a good inhibitory capacity against α-glucosidase, revealing its potential to be used as an antidiabetic agent, with inhibitory capacity approaching acarbose used to control type 2 diabetes *mellitus*. Contrarily, coffee HMWM showed poor antidiabetic capacity. Thus, instant chicory HMWM confer functional activities to chicory beverages. Nevertheless, in the case of the use of the whole roasted product, a balance between these positive effects and those not so positive that derive from Maillard reaction should be considered. In addition, the beneficial effects of the isolated melanoidins can be used for other food industry application, namely conferring antioxidant and antidiabetic properties, contributing also to the protection against pathogenic microorganisms.

## Figures and Tables

**Table 1:** General composition of the high molecular weight material (HMWM) of instant chicory and instant coffee.

Samples (HMWM)	Sugar (%, *w*/*w*)	Protein (%, *w*/*w*)	Phenolic (µg GAE ^1^/mg)	K_mix,405 nm_ (L/g/cm)	MBI
Chicory	28.9 ± 6.6 ^a^	5.7 ± 0.2 ^a^	60.0 ± 6.8 ^a^	1.3	1.9
Coffee	55.7 ± 7.0 ^b^	12.2 ± 0.7 ^b^	133.9 ± 17.7 ^b^	1.1	3.8

^1^ GAE—gallic acid equivalents. In each column, lowercase letters refer to significantly different values (*p* < 0.05) among samples.

**Table 2:** Phenolic compounds identified in the high molecular weight material of instant chicory and instant coffee.

Compounds (mg/g)	Chicory HMWM	Coffee HMWM
Esterified		
Caffeic acid	0.22 ± 0.03 ^a^	1.76 ± 0.03 ^b^
Ferulic acid	0.03 ± 0.00 ^a^	1.79 ± 0.04 ^b^
*p*-Coumaric acid	0.01 ± 0.00 ^a^	0.10 ± 0.00 ^b^
Glycosylated		
Caffeic acid	8.69 ± 0.07 ^a^	117.75 ± 6.76 ^b^
Ferulic acid	0.45 ± 0.03 ^a^	27.22 ± 1.20 ^b^
*p*-Coumaric acid	n.d. ^1^	1.18 ± 0.01
Condensed		
Catechol	7.12 ± 0.50 ^a^	13.31 ± 0.94 ^b^
Salicylic acid	3.13 ± 1.07 ^a^	1.29 ± 0.45 ^b^
2,3-Dihydroxybenzoic acid	2.81 ± 1.29 ^a^	8.79 ± 0.67 ^b^
Resorcylic acid	1.00 ± 0.35 ^a^	0.71 ± 0.10 ^a^
Gallic acid	2.78 ± 0.79	n.d.
Benzoic acid	3.39 ± 0.99 ^a^	4.19 ± 1.22 ^a^
3,4-Dihydroxybenzoic acid	19.02 ± 0.30 ^a^	86.66 ± 1.39 ^b^
4-Hydroxybenzoic acid	4.97 ± 0.84 ^a^	7.48 ± 1.27 ^a^
Gentisic acid	1.36 ± 0.05 ^a^	2.00 ± 0.08 ^b^

^1^ n.d.—not detected. Lowercase letters refer to significantly different values (*p* < 0.05) among samples.

**Table 3:** Antioxidant activity of chicory and coffee high molecular weight material (HMWM) determined in terms of IC_50_, T_IC50_, antiradical efficiency (AE) and ferric ion reducing capacity.

Samples (HMWM)	IC_50_ (mg/mL)	T_IC50_ (min)	AE (mg/mL/min)	Ferric Ion Reducing Capacity (µg Fe^2+^ eq ^1^/mg)
Chicory	0.28	47.05	5.95 × 10^−4^	11.34
Coffee	0.08	2.62 × 10^−11^	3.05 × 10^9^	23.46

^1^ Fe^2+^ eq—Fe^2+^ equivalents

## Data Availability

Data is contained within the article or Supplementary Materials.

## Supplementary Materials

The following supporting information can be downloaded at: https://www.mdpi.com/article/10.3390/foods12010134/s1, Figure S1: Kinetic behavior of chicory (purple color) and coffee (brown color) HMWM at the IC_50_ concentration in mg/mL, over time, expressed in terms of percentage of remaining ABTS^•+^, Table S1: Zone of inhibition (mm) of chicory and coffee HMWM against the Gram-positive *Staphylococcus aureus* and Gram-negative *Escherichia coli* bacteria.
