# Supplementary material for: Bioactive Properties of Instant Chicory Melanoidins and Their Relevance as Health Promoting Food Ingredients
Source: Foods. 2022 Dec 27;12(1):134. doi: 10.3390/foods12010134 (PMC9818759; doi:10.3390/foods12010134)
Supplement: Supplementary file 1 [file foods-12-00134-s001.zip › foods-2089042-supplementary.pdf]

Supplementary Materials

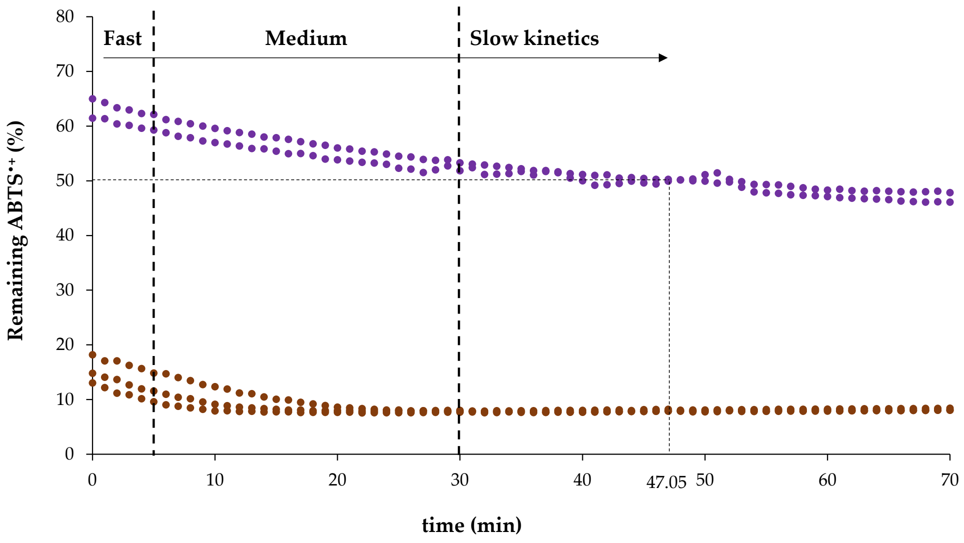

Figure S1. Kinetic behavior of chicory (purple color) and coffee (brown color) HMWM at the IC_{50} concentration in mg/mL, over time, expressed in terms of percentage of remaining $ABTS^{•+}$.

Table S1. Zone of inhibition (mm) of chicory and coffee HMWM against the Gram-positive *Staphylococcus aureus* and Gram-negative *Escherichia coli* bacteria.

		**Zone diameter (mm)**	
**Samples (Melanoidins)**	**Concentration (mg/mL)**	***S. aureus***	***E. coli***
Chicory	50	9	0
Coffee	50	14	0
**Positive controls**			
Amoxicillin		24	0
Penicillin		32	0
Ampicilina		33	0
Tetracycline		17	13
Ciprofloxacin		23	35

Amoxicillin, penicillin, ampicilina, tetracyline, and ciprofloxacin (all at 10 μg) were used as positive controls for Gram-positive and Gram-negative bacteria.
